# Use of Direct Acting Oral Anticoagulants in Elderly Patients with Atrial Fibrillation: A Multicenter, Cross-Sectional Study in Spain

**DOI:** 10.3390/jcm12031224

**Published:** 2023-02-03

**Authors:** Pablo Díez-Villanueva, Juan Cosín-Sales, Vanesa Roldán-Schilling, Vivencio Barrios, Diana Riba-Artés, Olga Gavín-Sebastián

**Affiliations:** 1 Cardiology Service, Hospital Universitario La Princesa, 28006 Madrid, Spain; 2 Cardiology Service, Hospital Arnau de Vilanova, 46015 Valencia, Spain; 3 Hematology Service, Hospital Universitario Morales Meseguer, 30008 Murcia, Spain; 4 Cardiology Service, Hospital Universitario Ramón y Cajal, 28034 Madrid, Spain; 5 Medical Affairs Department, Boehringer Ingelheim España, Sant Cugat del Vallés, 08173 Barcelona, Spain; 6 Hematology Service, Hospital Clínico Lozano Blesa, 50009 Zaragoza, Spain

**Keywords:** atrial fibrillation, elderly, frailty, non-vitamin K antagonist oral anticoagulants, inappropriate dosage, prescription monitoring

## Abstract

Non-vitamin K antagonist oral anticoagulants (NOACs) have substantially improved anticoagulation. However, data on NOAC use among elderly patients are scarce. We sought to describe NOAC use among elderly AF patients in Spain. We performed a non-interventional, multicenter, multispecialty, cross-sectional study in elderly (≥75 years) AF patients treated with NOACs for stroke prevention. Patients’ characteristics by NOAC treatment were compared using standardized differences (SDD). NOAC dosing was classified according to the Spanish summary of products characteristics (SmPC) into appropriate (recommended dose) and inappropriate (under and overdosed). Multivariate logistic regression analyses were used to explore factors associated with inappropriate dosing. 500 patients were included. Mean (SD) age was 81.5 (4.7) years, and 50% were women. Mean (SD) creatinine clearance was 57.4 mL/min (18.8), and 23.6% were frail. Dabigatran treatment totaled 38.4%, rivaroxaban 15.2%, apixaban 33.2%, and edoxaban 13.2%. Almost one-fourth of elderly patients treated with NOACs in Spain were inappropriately dosed (underdosing 14.4% and overdosing 9.6%). Underdosing was significantly associated with weight (OR = 1.03, 95%CI = 1.0–1.1), while higher a EHRA score decreased the risk of underdosing (OR = 0.47, 95%CI = 0.2–1.0). Overdosing was significantly associated with a history of ischemic stroke (OR = 2.95, 95%CI = 1.1–7.7). Addressing incorrect dosing among elderly AF patients is relevant to improve patient outcomes.

## 1. Introduction

Atrial fibrillation (AF) is the most frequent cardiac arrhythmia, and it is expected to affect over 17 million people in Europe by 2060 [1]. The risk of AF has been related to classical cardiovascular risk factors, although age is the most critical risk factor for AF [2,3]. The prevalence increases with age, reaching between 10% and 17% in those aged > 80 years [4]. AF is a major risk factor for stroke, causing substantial morbidity and mortality [5], especially in the elderly [6]. OAC reduces the risk of stroke and systemic embolism while improving survival in patients with AF. Non–vitamin K antagonist oral anticoagulants (NOACs) were developed as an alternative to vitamin K antagonists (VKA), showing a favorable risk-benefit profile compared to warfarin, since they reduce the risk of ischemic stroke and overall mortality and intracranial hemorrhage in the general population, especially in the elderly [7,8]. However, anticoagulation therapy in elderly patients with AF may be complex, since it may entail a higher bleeding risk [9]. Of note, randomized trials among elderly patients (75 years and older) reassure that NOACs are as effective as warfarin, or in some cases superior, with an overall improved safety profile while not requiring strict monitoring as VKA [6].

Current clinical guidelines on anticoagulation use in patients with AF recommend the use of NOAC, also in elderly patients [10]. However, several studies have shown that NOACs use in the elderly is deficient in clinical practice, since their use is low and underdosing or incorrect dosing is frequent [11,12,13]. In turn, these practice may lead to more adverse events [14].

In addition to age, other baseline characteristics and comorbidities should be accounted for when considering anticoagulation treatment. Frailty, a biologic syndrome manifested as a decreased ability to recover from stressors due to a cumulative decline in the individuals’ physiological systems, homeostatic reserves, and resiliency, is highly prevalent in patients with AF [15,16]. Older frail adults have a higher risk for stroke and mortality, yet are less likely to be treated with oral anticoagulants, despite obtaining similar benefits from anticoagulation compared with those without frailty [17]. Furthermore, if anticoagulated, they are more likely to receive warfarin than NOACs [18,19]. This underscores the need for a critical geriatric assessment and patient profile, including age, frailty, and comorbidities when prescribing oral anticoagulation [20,21,22].

While NOAC use remains challenging among the elderly, effective strategies should be developed to improve clinical outcomes in elderly AF patients. The objective of this study was to describe the characteristics of elderly (≥75 years old) AF patients treated with NOACs in Spain, as well as current patterns and management of this therapy.

## 2. Materials and Methods

### 2.1. Study Design

A non-interventional, multicenter, multispecialty, and cross-sectional study was conducted in AF elderly patients with ongoing treatment with NOACs for stroke prevention. Each patient’s therapeutic strategy was determined before the study visit by the investigator, and participation in the study did not interfere with the physician’s prescription habits. The study required a single visit coinciding with patients’ routine follow-up visits. Data collection took place without interfering with the regular clinical visit.

### 2.2. Setting

A total of 503 patients were consecutively recruited to the study between September 2019 and August 2020 from 36 sites. Site selection contemplated several geographical areas according to the distribution of the overall population to ensure a nationwide representative sample. In addition, sites were selected based on different levels of medical care (hospital setting, private consultations, specialty medical offices, and nursing homes), and a feasibility questionnaire was used to determine that both sites and investigators were qualified to meet the study needs, in compliance with the applicable regulatory requirements.

### 2.3. Subjects

The eligibility criteria included age ≥ 75 years, prior diagnosis of AF, ongoing treatment with NOACs for at least three months, and NOAC treatment for approved indications (according to each drugs’ Summary of Product Characteristics (SmPC). Exclusion criteria included moderate/severe mitral stenosis or mechanical prosthetic valves, current participation in a clinical trial, and any contraindication for NOAC treatment according to the SmPC.

### 2.4. Data Collection

At the study visit, baseline clinical characteristics were collected, including traditional cardiovascular risk factors and socio demographic information, as well as history of previous thromboembolic and bleeding events. Frailty was assessed with the Clinical Frailty Scale (CFS) using the modified Rockwood scale [20,23]. AF related symptoms were evaluated with the Modified EHRA scale [24]. Additionally, the New York Heart Association (NYHA) classification of heart failure, the CHA_2_DS_2_-VASc, and the HAS-BLED scales’ scores were calculated for each patient. Comorbidity was assessed by the age-adjusted Charlson Comorbidity Index (CCI) [25]. In addition, investigators reviewed the patient’s medical records to retrieve other information needed to address the study objectives, including antiplatelet, or anticoagulant treatments. Investigators were required to answer the reasons for NOAC initiation (at the time of the first NOAC prescription) in terms of the Spanish health authorities’ recommendations for NOAC initiation (therapeutic positioning report). Three categories for NOAC initiation were considered: (1) clinical reasons, (2) situations related to International Normalized Ratio (INR) control, and (3) other. CFS scores were categorized into: Frailty (CFS > 4) and non-frailty (CFS ≤ 4).

The appropriateness of NOAC dosing was analyzed based on Spanish health authorities’ recommendations (therapeutic positioning report). The standard dose for each NOAC was 150 mg twice daily for dabigatran, 20 mg once daily for rivaroxaban, 5 mg twice daily for apixaban, and 60 mg once daily for edoxaban. Dose reduction criteria are specific to each NOAC and dependent on certain patient characteristics, including age, weight, serum creatinine level, creatinine clearance, and concomitant medications. The adherence with labeled dosing of each NOAC in each study patient was evaluated based on each drug’s SmPC. Patients were classified as appropriately (prescription of the recommended dose) or inappropriately (prescription not in line with recommendations) dosed according to the current NOAC dose, based on the recommended dosages for each drug in the SmPC (Table S1). Inappropriate dosing included both underdosed patients and overdosed patients. For dabigatran, overdosing or underdosing should not really be considered as such, but as an appropriateness of doses recommended in the SmPC, because both doses were randomized in the RELY clinical trial [26].

In patients with previous history of thromboembolic and bleeding events, we recorded if patients were ongoing antiplatelet or antithrombotic treatment at the time of the event. Treatment type (NOAC, VKA, and antiplatelet treatments) at the time of the event was also recorded.

### 2.5. Statistical Analysis

All variables were described with measures of central tendency (mean and median), variability/dispersion (standard deviation (SD), and interquartile ranges (IQR) and ranges) for continuous variables and distributions of absolute and relative frequencies for categorical variables. Patient characteristics by NOAC type were compared by obtaining standardized differences (SDD) between dabigatran and other NOACs. According to Cohen, to quantify the magnitude of difference between groups on baseline variables an effect size index for the comparison of two sample means can be used and interpreted as “a measure of the average difference between means expressed in standard deviation units” [27]. Effect Size Indices of 0.2, 0.5, and 0.8 can be used to represent small, medium, and large effect sizes, respectively. In the statistical analysis, we have used the standardized difference (d Cohen), frequently used in epidemiology since it better quantifies the size of the difference between two groups than other methods. In our study, the standardized difference with absolute value < 0.2 was considered as balance between groups. Standardized differences between dabigatran and other NOACs has been estimated for all covariates, with a minimum of 50 patients required for each group. Therefore, those values lower than −0.2 or higher than +0.2 were considered as a difference between groups, those being higher absolute values, and greater difference in the variables. Cohen suggested that Effect Size Indices of 0.2, 0.5, and 0.8 can be used to represent small, medium, and large effect sizes, respectively [28,29].

The pattern of usage of NOAC was described as the percentage of patients by NOAC type and dose, reasons for NOAC initiation (in terms of prevention and according to SmPC), and treatment switches. The percentage of appropriately and inappropriately dosed patients (including underdosed and overdosed) was described for current NOAC treatment. Treatments received at the time of thromboembolic and bleeding events were described, including NOAC, VKA, and antiplatelet treatments. Univariate and multivariate logistic regression analyses were performed to explore factors associated with NOAC under and overdosing. Variables included in the multivariate regression analysis and the final regression model were selected based on the investigator’s knowledge and the available literature, which guided variable selection according to their potential of influencing the investigated association. All analyses were conducted using SAS, version 7.15.

## 3. Results

### 3.1. Population Baseline Characteristics

A total of 503 patients were enrolled, of which 3 (0.6%) were excluded because they did not fulfill the selection criteria, leaving 500 eligible patients. The mean (SD) age was 81.5 (4.7), and 50% were female (*n* = 250). The mean (SD) creatinine clearance value was 57.4 mL/min (18.8). Current NOAC treatment was 38.4% for dabigatran (192 patients), 15.2% rivaroxaban (76 patients), 33.2% apixaban (166 patients), and 13.2% edoxaban (66 patients). Other baseline characteristics are shown in Table 1.

Mean (SD) time since AF diagnosis was 5.5 (5.3) years. Most patients (*n* = 227, 47%) had permanent AF, followed by paroxysmal AF (*n* = 131, 27.1%). According to EHRA scores, 45% had mild symptoms, 20% had moderate symptoms, and 5.4% had severe or disabling symptoms.

The most prevalent comorbidities were hypertension (82.4%, *n* = 412), heart failure (36.8%, *n* = 184), and diabetes (30.8%, *n* = 154) (Table 2). Mean (SD) CHA2DS2-VASc score was 4.3 (1.4). A HAS-BLED score ≥3 was observed in 24.6% (*n* = 123) (Table 2).

A total of 45.8% had a caregiver, and the overall proportion of patients living at nursing homes was low (3.1%). Overall frailty prevalence calculated using the clinical frailty scale (CFS > 4) was 23.6%, which was lowest for dabigatran (17.2%), followed by edoxaban (21.2%) and rivaroxaban (27.6%), while highest for apixaban (30.1%) (Figure S1).

### 3.2. Patient Characteristics according to NOAC Treatment

Table 1 shows SDD of baseline characteristics and laboratory values by NOAC group. In general, a higher proportion of patients treated with dabigatran were aged 75–79 years (48.4%, SDD = 0.26925), of male sex (59.9%, SDD =−0.32575), and had a higher creatinine clearance with a mean (SD) of 63.5 mL/min (18.5) (SDD = −0.49546) compared to the other NOACs.

The distribution of comorbidities was similar across NOACs (Table 2). Dabigatran patients had a lower mean (SD) CCI of 5.3 (1.7) (SDD = 0.33904), while edoxaban patients had the highest CCI, at 6.3 (2.4). The lowest CHA2DS2-VASc score was observed in dabigatran patients (mean 4.1, SD 1.3), while apixaban had the highest score (mean 4.6, SD 1.4) (SDD = 0.3478) (Table 2).

### 3.3. Previous Thromboembolic and Bleeding Events

Treatment at the time of the previous thromboembolic and bleeding events is shown in Table 3. A total of 190 previous thromboembolic events occurred within the sample, affecting 25.2% of patients. The most frequent prior thromboembolic event was ischemic stroke (*n* = 51, 10.3%). Previous bleeding events were present in 15.5% of patients, among which a total of 123 events were reported, being digestive bleeding the most frequent (*n* = 40, 8.0%). While most thromboembolic events occurred while patients were untreated, a high proportion of bleeding events (49.6%) occurred while patients were treated with NOACs. A total of 13.7% thromboembolic events and 30.9% bleeding events occurred while patients were treated with VKA.

### 3.4. NOAC Prescription Patterns: Indications, Dose Appropriateness, and Treatment

Most patients initiated NOACs for primary prevention (78.1%), and secondary prevention amounted to 21.9%. The underlying reasons for NOAC prescription (based on the Spanish health authorities’ recommendations) were situations related to INR control in 50.2% (*n* = 251), followed by other reasons in 41% (*n* = 205) (physician-patient agreement in 94.3%), and clinical reasons in 8.8% (*n* = 44) (high risk of intracranial hemorrhage in 40.9%, followed by hypersensitivity or contraindication to VKA in 31.8%).

**Figure 1:**
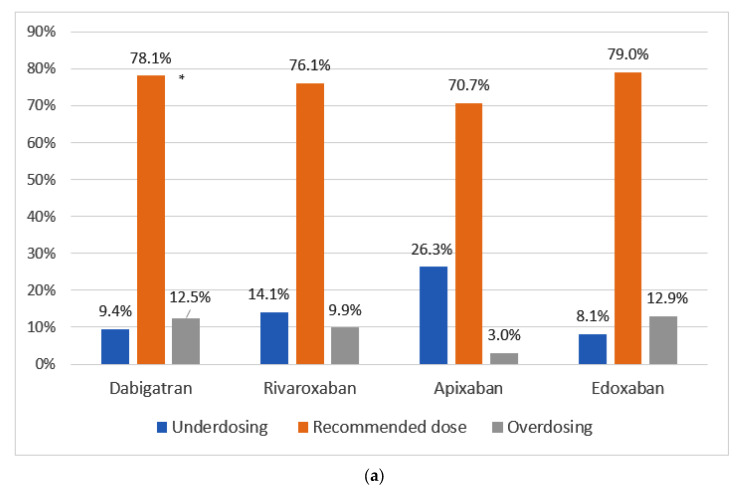

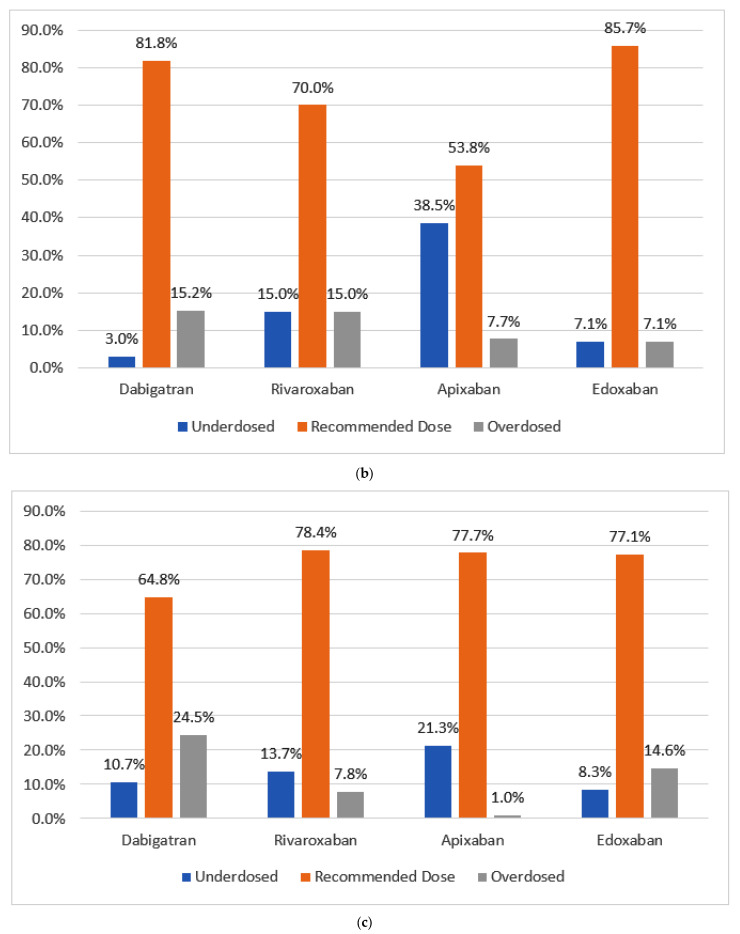
Appropriateness of NOAC dosing by NOAC type according to SmPC recommendations in the (**a**) overall population, in (**b**) patients with frailty, and in (**c**) patients without frailty. Appropriate dose according to the SmPC. Inappropriate dose includes under and overdosed patients, according to the SmPC recommendations. Frailty was defined as CFS > 4. *: For dabigatran, underdosed/overdosed should not really be considered as such, but as an appropriateness of doses recommended in the SmPC, because both doses were randomized in the RE-LY clinical trial.

The proportion of patients receiving the recommended NOAC dose according to SmPC was 76% (*n* = 380). Among inappropriate dosed patients (24%), underdosing and overdosing totaled 14.4% and 9.6%, respectively. The proportion of adequately treated patients by NOAC type ranged from 70.7% apixaban to 79% edoxaban (Figure 1a). The proportion of underdosing ranged between 8.1%, 9.4%, and 14.1% among patients treated with edoxaban, dabigatran, and rivaroxaban, respectively, and reached 26.3% for apixaban. Overdosing was observed in 3% of patients with apixaban, 9.9% of patients with rivaroxaban, 12.5% of dabigatran patients, and 12.9% of patients with edoxaban. When the appropriateness of NOAC doses was assessed among frail patients, the proportion of patients with appropriate doses was higher for dabigatran and edoxaban when compared to the overall population, while slightly lower for rivaroxaban. This differed considerably for apixaban, among which the proportion of frail patients treated with an adequate NOAC dose was substantially lower than the overall population (Figure 1b). In contrast, the percentage of adequately treated among non-frail was lowest for dabigatran (64.8%), while similar among the rest of NOACs (Figure 1c). One caveat when considering appropriateness of doses for dabigatran, is that overdosing or underdosing should not really be considered as such, but as an appropriateness of doses recommended in the SmPC, because both doses were randomized in the RELY clinical trial [26]. Additional details related to NOAC treatment dose by sex is showed in Table S2.

**Figure 2:**
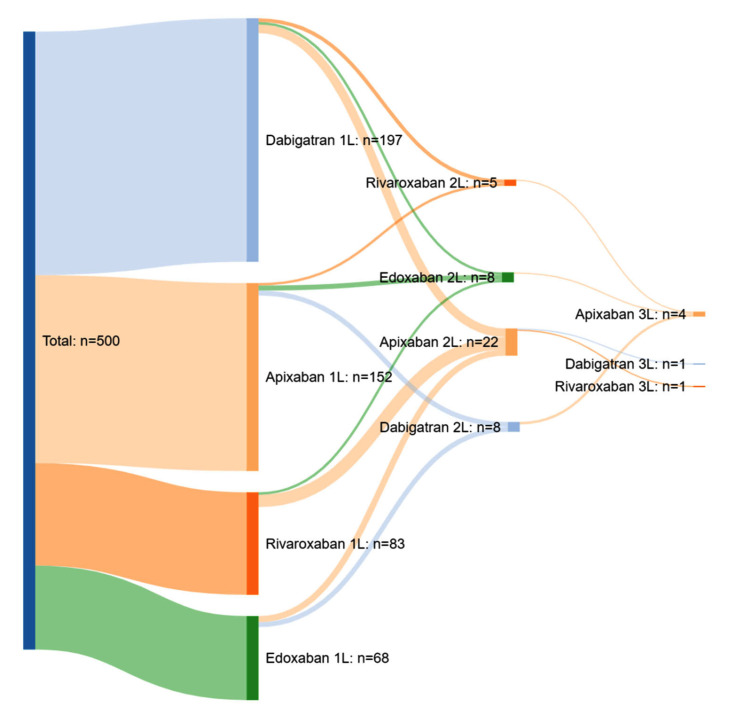
Sankey diagram of NOAC treatment changes. 1L = 1st Line. 2L = 2nd Line (one NOAC switch). 3L = 3rd Line (two NOAC switches). Line refers to the NOAC used prior to switching to the following NOAC.

Overall, the mean (SD) time since NOAC treatment initiation was 2.32 (2.02) years. During this period, a total of 8.6% of patients (*n* = 43) switched NOACs (one switch in 7.4%, two switches in 1.2%), and most switches were to apixaban (Figure 2). The main reasons for switching NOACs were adverse events (49%), followed by the investigator’s decision (34.7%).

### 3.5. Factors Influencing under and Overdosing

**Figure 3:**
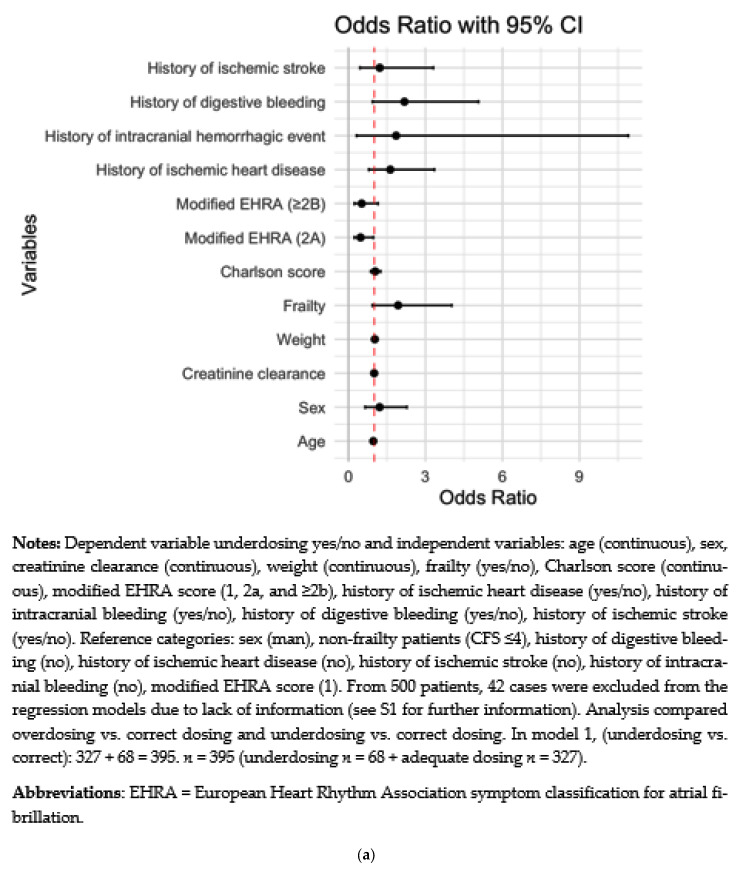

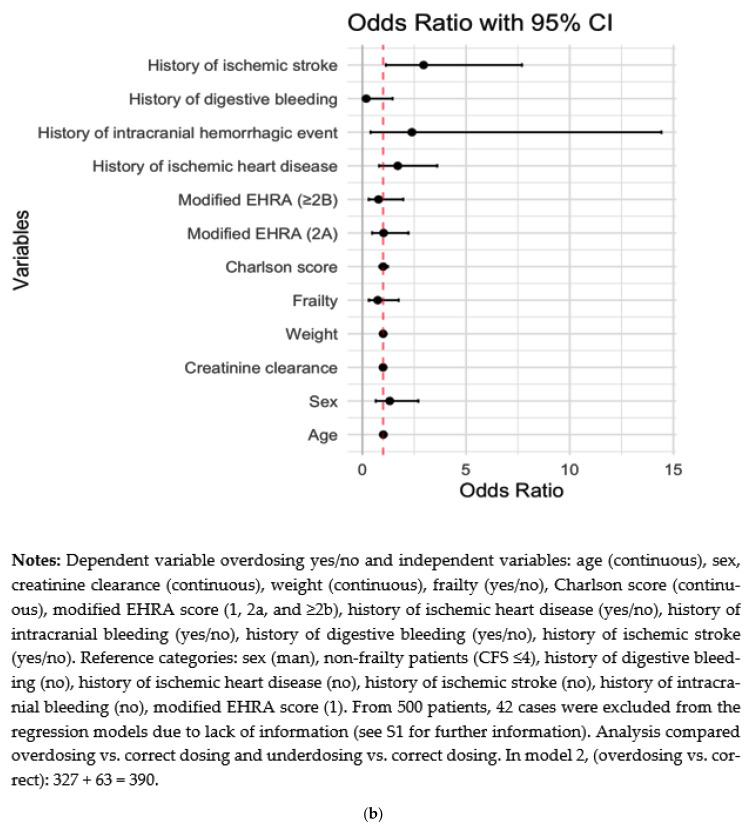
Forest plot of variables influencing NOAC (**a**) underdosing and (**b**) overdosing.

A higher risk of underdosing, according to the SmPC, was significantly associated in the multivariable analysis with weight (OR = 1.03, 95%CI = 1.0–1.1), while a modified EHRA score of 2A decreased the risk of underdosing (OR = 0.47, 95%CI = 0.2–1.0), and a similar trend was observed for EHRA scores ≥ 2B (OR = 0.52, 95%CI = 0.2–1.2) (Figure 3a). While frailty was not significantly associated with underdosing, a trend towards underdosing was noted among frail patients (OR = 1.93, 95%CI = 0.9–4.0). Similarly, a history of digestive bleeding also showed a trend towards underdosing (OR = 2.21, 95%CI = 0.9–5.1).

In terms of NOAC overdosing (Figure 3b), a previous history of ischemic stroke was significantly associated with overdosing (OR = 2.95, 95%CI = 1.1–7.7).

## 4. Discussion

Anticoagulation is the most effective therapy to prevent stroke and other thromboembolic events in individuals with AF. However, treating elderly patients can be challenging because of increasing comorbidities and polypharmacy, adding contraindications or drug interactions. Concerns of increased bleeding risk can often lead to underuse or underdosing of NOACs in elderly patients. This study describes the current use of NOACs among elderly patients (≥75 years old) with AF without moderate/severe mitral stenosis or mechanical prosthetic valves in a nationwide sample in Spain.

Patients included in our sample represent an elderly and complex population, as shown by the proportion of patients aged ≥ 80 (58%), the high prevalence of heart failure (36.8%) and diabetes (30.8%), the increased risk of stroke (mean CHA_2_DS_2_-VASc score 4.3), and the proportion of patients with HAS-BLED score ≥ 3 (24.6%).

Reduced NOAC dose was observed in 47% of patients in our study, varying slightly according to NOAC type. This finding is in line with a study from the UK reporting NOAC reduced dosing among 38% of new users aged ≥ 70, while in 54% of those aged ≥ 80 [30]. Regarding the adequateness of NOAC dosing as recommended by the SmPC, 76.0% were appropriately dosed, while 24% were inappropriately dosed. Among inappropriately dosed patients, most were underdosed (60%). Our results align with a recent study among elderly patients in the US, which reported that 23% of patients were inappropriately dosed, of which most were underdosed (78.0%) [31]. Other studies have identified a higher proportion of suboptimal dosing among patients aged ≥ 90, reported at 41.5% [32,33].

We identified weight and EHRA score as predictors of underdosing. Weight is a determining factor for the dosing of NOACs [34]. In our study, a higher EHRA score was found to protect from underdosing. We found patients with lower EHRA score to be less often women, with lower weight, frailty and comorbidities, which in turn have been associated with lower adequate NOAC [35].

Regarding frailty, some previous studies have shown that the rate of OAC prescription is lower in frail elderly patients as compared to non-frail patients [31,36]. In our study, on the other hand, a non-significant trend towards underdosing was noted among frail patients. It is important to note that in these fragile patients, adequate OAC is associated to lower adverse clinical outcomes [37]. Finally, a trend towards underdosing was observed among patients with a digestive bleeding.

In terms of factors associated with overdosing, we identified history of ischemic stroke to be significantly associated with overdosing. However, guideline-discordant therapy NOAC dosing might increase the risk of stroke and bleeding [38]. One consideration to be made in the case of dabigatran is that overdosing or underdosing should not be considered as such, but as appropriate doses recommended in the SmPC, because both doses were randomized in the RELY clinical trial [26]. Additionally, it must be noted that a once-daily low dose of edoxaban (15 mg) in elderly Japanese patients, was superior to placebo in preventing stroke or systemic embolism without significantly increasing the risk of bleeding [39]. However, it remains to be seen if these results are applicable to other populations. Further studies should investigate the physicians’ perspectives motivating guideline-discordant doses.

It is worth mentioning that 69 patients had missing information on creatinine clearance, which is relevant since poor renal function is associated with older age and worse clinical outcomes. While NOAC dose adjustment depends on specific comorbidities and laboratory values, creatinine clearance affects almost all NOACs. Missing essential information to calculate creatinine clearance has also been noted in previous studies [11]. Additionally, about 20% of patients had missing values of liver enzymes, which is also concerning since NOACs are not recommended in the presence of severe hepatic impairment. Missing liver function tests have also been noted in a previous study [40].

The mean time since the first NOAC treatment initiation was 2.32 years, and 8.6% of patients switched treatment over this period. Previous data from the literature on switching are variable and likely conditioned by follow-up time [41,42,43]. Within our sample, most switches were to apixaban. However, the percentage of apixaban-treated patients with inadequate doses was higher compared to other NOACs, even more so when only frail patients were considered. The prevalence of frailty within our study was 23.6%. The proportion of frail patients with appropriate NOAC dosing was higher for dabigatran and edoxaban than the overall population, while slightly lower for rivaroxaban. However, among frail patients treated with apixaban, the proportion of patients adequately dosed was considerably lower. Based on these findings, we hypothesize that physicians’ perceptions of frailty may differ from instrument-based frailty scores or that other underlying reasons incline physicians to prescribe a reduced NOAC dose. The prevalence of frailty among patients with AF is highly variable, ranging from 4.4–75.4%, and probably related to differences in the screening instruments used and varying frailty awareness [16,44]. Lower OAC use has been noted among frail AF patients at hospital admission, which likely reflects real community prescription patterns [45]. However, increasing frailty awareness among professionals is relevant, since AF patients with frailty have a greater risk of cardio-embolic stroke than those without frailty [46], and frailty has also been associated with higher mortality in AF patients [46,47,48]. Furthermore, OAC treatment has been reported to reduce the risk of ischemic stroke, major bleeding, or cardiovascular death risk in frail AF individuals aged ≥ 65 among Asian patients [49]. In addition, all NOACs showed a lower incidence of stroke, bleeding, and mortality compared to warfarin [49]. This finding is highly relevant, considering NOACs are less often used among AF patients with frailty [18,19]. However, one caveat to this study is that the Hospital Frailty Risk Score calculated using administrative claim data were used to identify frailty, which may have underestimated frailty among older people with few or no past hospital visits [49]. Further evidence on how to best manage frail and elderly AF patients, especially in populations of European descent, is warranted.

### Limitations

Some limitations are inherent to this study design, which must be acknowledged. Some data used for this study relied on the data extracted from clinical records, which are subject to missing values. Dabigatran-treated patients were overrepresented in our sample (38.4%), which may not represent actual prescription patterns. However, patient-level selection bias was minimized by enrolling consecutive patients. Still, dabigatran treated patients could have been more inclined to participate. Patients included in this study may not reflect all patients who initiate NOAC, since a minimum 3-month treatment time was required. Patients with a higher visit frequency were more likely to be enrolled and could be overrepresented in the sample. Recruitment for this study was affected by COVID-19, during which recruitment was suspended given the epidemiological situation. This may have imposed a selection bias (and even survival) on our sample. Channeling bias (a form of confounding when a drug is preferentially prescribed to patients with different baseline characteristics) was assessed using standardized differences. HAS-BLED and CHA2DS2-VASc scores were assessed at the time of the study visit. Therefore, it is unattainable to judge whether investigators took these scores into account during routine practice and NOAC prescription. In patients with prior thromboembolic and bleeding events, we recorded if they were ongoing treatment at the time of the event and treatment type. However, the adequacy of dosing or patient adherence at the time of the event was not assessed.

## 5. Conclusions

This study describes a large multicenter sample of AF patients aged ≥ 75 treated with NOACs in Spain and provides a first estimation of prescription practice by professionals from multiple specialties. The prevalence of frailty within our sample was high. The considerable comorbidity profile and the high risk of stroke outline the complexity of this patient population and enlightens the need to assess and treat elderly patients with AF correctly. Within our sample, we did not observe significant differences in over or underdosing among patients with frailty, although we observed a trend towards the latter in frail patients. Our results can be used to optimize patient care, especially regarding the need to address incorrect dosing among elderly patients and reinforce consideration of laboratory parameters that could prove harmful when left unaccounted.

## Figures and Tables

**Table 1:** Baseline sociodemographic characteristics and laboratory values according to current NOAC type.

	Dabigatran (*n* = 192)	Rivaroxaban (*n* = 76)	Apixaban (*n* = 166)	Edoxaban (*n* = 66)	Total	SDD
Age (years)						
Mean (SD)	80.83 (4.50)	80.89 (4.64)	82.29 (4.90)	82.02 (4.80)	81.48 (4.73)	0.22552
75–79 years	93 (48.4%)	34 (44.7%)	59 (35.5%)	24 (36.4%)	210 (42.0%)	0.26925
80–84 years	59 (30.7%)	23 (30.3%)	48 (28.9%)	22 (33.3%)	152 (30.4%)	
≥85 years	40 (20.8%)	19 (25.0%)	59 (35.5%)	20 (30.3%)	138 (27.6%)	
Sex						
Female	77 (40.1%)	46 (60.5%)	94 (56.6%)	33 (50.0%)	250 (50.0%)	−0.32575
BMI (kg/m^2^)						
Mean (SD)	28.58 (4.06)	28.13 (4.77)	28.32 (4.71)	27.46 (3.88)	28.27 (4.42)	−0.10793
Smoking habit						
Smoker	6 (3.3%)	1 (1.3%)	4 (2.5%)	1 (1.5%)	12 (2.5%)	0.24214
Alcohol consumption						
Abuse	0	0	1 (0.7%)	1 (1.7%)	2 (0.4%)	0.16657
Creatinine clearance						
Mean (SD)	63.50 (18.49)	55.42 (17.59)	54.45 (18.65)	53.04 (18.40)	57.37 (18.83)	−0.49546
Creatinine clearance—range						
<15	0	0	0	0	0	0.48605
15–29	0	3 (4.2%)	10 (6.4%)	7 (11.1%)	20 (4.6%)	
30–59	72 (51.1%)	41 (57.7%)	92 (59.0%)	37 (58.7%)	242 (56.1%)	
60–89	55 (39.0%)	25 (35.2%)	46 (29.5%)	18 (28.6%)	144 (33.4%)	
≥90	14 (9.9%)	2 (2.8%)	8 (5.1%)	1 (1.6%)	25 (5.8%)	
AST (UI/L)						
Median (Q1–Q3)	21.0 (18.0; 28.0)	20.5 (17.0; 25.0)	21.0 (16.0; 29.0)	19.0 (14.0; 24.0)	21.0 (17.0; 27.0)	−0.04836
ALT (UI/L)						
Median (Q1–Q3)	19.0 (13.0; 25.0)	16.0 (13.0; 21.0)	16.0 (12.0; 25.0)	14.5 (11.0; 21.0)	16.5 (12.0; 24.0)	−0.1275
Total bilirubin (mg/dl)						
Median (Q1–Q3)	0.7 (0.5; 1.0)	0.7 (0.4; 0.9)	0.6 (0.5; 0.8)	0.6 (0.5; 1.0)	0.7 (0.5; 0.9)	−0.15063
Hemoglobin (g/dl)						
Median (Q1–Q3)	13.1 (12.0; 14.4)	13.1 (12.3; 14.5)	13.1 (11.8; 14.3)	13.4 (12.3; 14.5)	13.2 (12.1; 14.4)	−0.06259
Platelet levels (×10^3^/μL)						
Median (Q1–Q3)	197.5 (156.0; 234.0)	209.9 (176.0; 254.0)	200.0 (160.5; 246.9)	179.5 (151.5; 223.0)	198.0 (160.0; 241.0)	0.11675

SDD = Standardized differences (Dabigatran vs. other NOACs). ALT = Alanine Transaminase. AST = Aspartate Aminotransferase. BMI= body mass index. Some categories had missing values: BMI, missing *n* = 126; creatinine clearance, missing *n* = 69; smoking habit, missing *n* = 14; alcohol consumption, missing *n* = 52; AST, missing *n* = 113; ALT, missing *n* = 102; Hemoglobin, missing *n* = 15; platelet levels, missing *n* = 29. Standardized differences were obtained comparing Dabigatran vs. other NOACs. Creatinine clearance is expressed by mL/min/1.73 m^2^.

**Table 2:** Distribution of comorbidities and clinical scores according to NOAC type.

	Dabigatran (*n* = 192)	Rivaroxaban (*n* = 76)	Apixaban (*n* = 166)	Edoxaban (*n* = 66)	Total	SDD
**Comorbidities**						
Heart failure						
*n* (%)	63 (32.8%)	22 (28.9%)	70 (42.2%)	29 (43.9%)	184 (36.8%)	0.13513
Hypertension						
*n* (%)	146 (76.0%)	63 (82.9%)	144 (86.7%)	59 (89.4%)	412 (82.4%)	0.26653
Coronary artery disease						
*n* (%)	35 (18.3%)	13 (17.1%)	21 (12.9%)	13 (19.7%)	82 (16.5%)	−0.0779
Myocardial infarction						
*n* (%)	16 (8.3%)	11 (14.5%)	18 (10.9%)	11 (16.7%)	56 (11.2%)	0.15248
Peripheral vascular disease						
*n* (%)	20 (10.4%)	5 (6.7%)	15 (9.0%)	7 (10.6%)	47 (9.4%)	−0.05506
Cerebrovascular disease						
*n* (%)	39 (20.3%)	13 (17.1%)	33 (19.9%)	9 (13.6%)	94 (18.8%)	−0.06251
Dementia						
*n* (%)	4 (2.1%)	4 (5.3%)	13 (7.8%)	6 (9.1%)	27 (5.4%)	0.25452
COPD						
*n* (%)	31 (16.1%)	10 (13.2%)	24 (14.5%)	12 (18.2%)	77 (15.4%)	−0.03206
Diabetes mellitus						
Uncomplicated, *n* (%)	48 (25.0%)	18 (23.7%)	36 (21.7%)	11 (16.7%)	113 (22.6%)	0.22544
End-organ damage, *n* (%)	9 (4.7%)	7 (9.2%)	17 (10.2%)	8 (12.1%)	41 (8.2%)	
Chronic kidney disease						
*n* (%)	26 (13.5%)	12 (15.8%)	36 (21.7%)	18 (27.3%)	92 (18.4%)	0.20877
**Clinical risk Scores**						
Charlson Comorbidity index score						
Mean (SD)	5.3 (1.7)	5.4 (1.6)	6.0 (2.0)	6.3 (2.4)	5.7 (2.0)	0.33904
CHA_2_DS_2_-VASc score						
Mean (SD)	4.1 (1.3)	4.4 (1.3)	4.6 (1.4)	4.3 (1.3)	4.3 (1.4)	0.3478
HAS-BLED score						
Mean (SD)	2.0 (0.8)	1.6 (0.8)	2.0 (0.9)	2.1 (0.9)	2.0 (0.9)	−0.07326
HAS-BLED (risk of bleeding)						
Low risk (score 0), *n* (%)	0	0	0	0	0	
Intermediate risk (score 1–2), *n* (%)	147 (76.6%)	64 (84.2%)	118 (71.1%)	48 (72.7%)	377 (75.4%)	
High risk (score ≥ 3), *n* (%)	45 (23.4%)	12 (15.8%)	48 (28.9%)	18 (27.3%)	123 (24.6%)	
Frailty (CFS scoring > 4)	33 (17.2%)	21 (27.6%)	50 (30.1%)	14 (21.2%)	118 (23.6%)	

SDD = Standardized differences (Dabigatran vs. other NOACs). COPD = Chronic obstructive pulmonary disease. Charlson comorbidity index was age-adjusted. Some categories had missing values: coronary artery disease, missing *n* = 4; myocardial infarction, missing *n* = 1; COPD, missing *n* = 1; TIA, missing = 6; ischemic stroke, missing *n*= 5; history of bleeding events, missing 2; digestive bleeding, missing *n* = 2; Charlson comorbidity index, missing *n* = 2. CFS: Clinical Frailty Scale.

**Table 3:** Distribution of previous thromboembolic and bleeding events by ongoing treatment at the time of the event.

Prior Thromboembolic and Bleeding Events/Treatment at the Time of the Event	Total Events	Untreated	Antiplatelets	VKA	NOAC	VKA + Antiplatelet	NOAC + Antiplatelet
Thromboembolic events, *n* (%)	190 (100.0%)	98 (51.6%)	42 (22.1%)	26 (13.7%)	17 (8.9%)	4 (2.1%)	3 (1.6%)
Bleeding events, *n* (%)	123 (100.0%)	14 (11.4%)	7 (5.7%)	38 (30.9%)	61 (49.6%)	0 (0.0%)	3 (2.4%)

VKA = Vitamin K antagonists. NOAC = Non-vitamin K antagonist oral anticoagulants.

## Data Availability

To ensure independent interpretation of clinical study results and enable authors to fulfill their role and obligations under the ICMJE criteria, Boehringer Ingelheim grants all external authors access to clinical study data pertinent to the development of the publication. In adherence with the Boehringer Ingelheim Policy on Transparency and Publication of Clinical Study Data, scientific and medical researchers can request access to clinical study data when it becomes available on Vivli—Center for Global Clinical Research Data, and earliest after publication of the primary manuscript in a peer-reviewed journal, regulatory activities are complete, and other criteria are met. Please visit Medical & Clinical Trials|Clinical Research|MyStudyWindow for further information.

## Supplementary Materials

The following supporting information can be downloaded at: https://www.mdpi.com/article/10.3390/jcm12031224/s1, Figure S1: Clinical Frailty Scale score at the time of the study visit according to current NOAC type; Table S1: Recommended dosing criteria for each NOAC according to the SmPC; Table S2: Current NOAC treatment dose by sex.
