# Supplementary material for: Use of Direct Acting Oral Anticoagulants in Elderly Patients with Atrial Fibrillation: A Multicenter, Cross-Sectional Study in Spain
Source: J Clin Med. 2023 Feb 3;12(3):1224. doi: 10.3390/jcm12031224 (PMC9917412; doi:10.3390/jcm12031224)
Supplement: Supplementary file 1 [file jcm-12-01224-s001.zip › jcm-2104344-supplementary.pdf]

SUPPLEMENTARY MATERIAL

Table S1. Recommended dosing criteria for each NOAC according to the SmPC

Figure S1. Clinical Frailty Scale score at the time of the study visit according to current NOAC type

Table S2. Current NOAC treatment dose by sex

Table S1. Recommended dosing criteria for each NOAC according to the SmPC

NOAC	Standard dose	Reduced dose	SmPC recommendations for dose reduction
Dabigatran	150mg BID	110mg BID	Patients with the following criteria:<br>• age ≥ 80 years or concomitant use of verapamil*<br>• age < 80 years with high bleeding risk (evaluated as HAS-BLED ≥ 3) consider low dose with at least one of the following criteria:<br>o age between 75–79 years<br>o moderate renal impairment (CrCl 30– 50 mL/min)<br>o presence of gastritis, esophagitis or gastroesophageal reflux*
Rivaroxaban	20mg qD	15mg qD	Patients with the following criteria:<br>• moderate/severe renal impairment (CrCl 15–49 ml/min)
Apixaban	5mg BID	2.5 mg BID	Patients with the following criteria:<br>• severe renal impairment (CrCl 15–29 mL/min)<br>• moderate renal impairment (CrCl ≥ 30 mL/min) and at least comply 2 of 3 following criteria:<br>o age ≥ 80 years<br>o body weight ≤ 60 kg<br>o serum creatinine ≥ 1.5 mg/dL (133 μmol/L)
Edoxaban	60mg qD	30mg qD	Patients meeting one of the following criteria:<br>• moderate/severe renal impairment (CrCl 15–50 ml/min)<br>• body weight ≤ 60 kg<br>• concomitant use of the P-gp inhibitors (dronedarone, ciclosporin, erythromycin, ketoconazole) *

BID: twice a day; qD: once a day; CrCl: creatinine clearance. SmPC: Summary of product characteristics. * Criteria not evaluated in the present study. According to table S1, the recommended dose for some treatments relies on the fulfilment of at least one of the criteria. Therefore, if a patient met one criteria, despite having CrCl not available, the patient could be assessed in terms of dosing adequateness. For example, if a patient taking Edoxaban weighed <60 kg and was receiving a dose >30 mg, this dose should have been reduced and considered for analysis, even though CrCl was not available. Within our sample, only 42 cases (8.4%; 5 with Rivaroxaban, 33 with Apixaban and 4 with Edoxaban) could not be analyzed in terms of dosing adequateness.

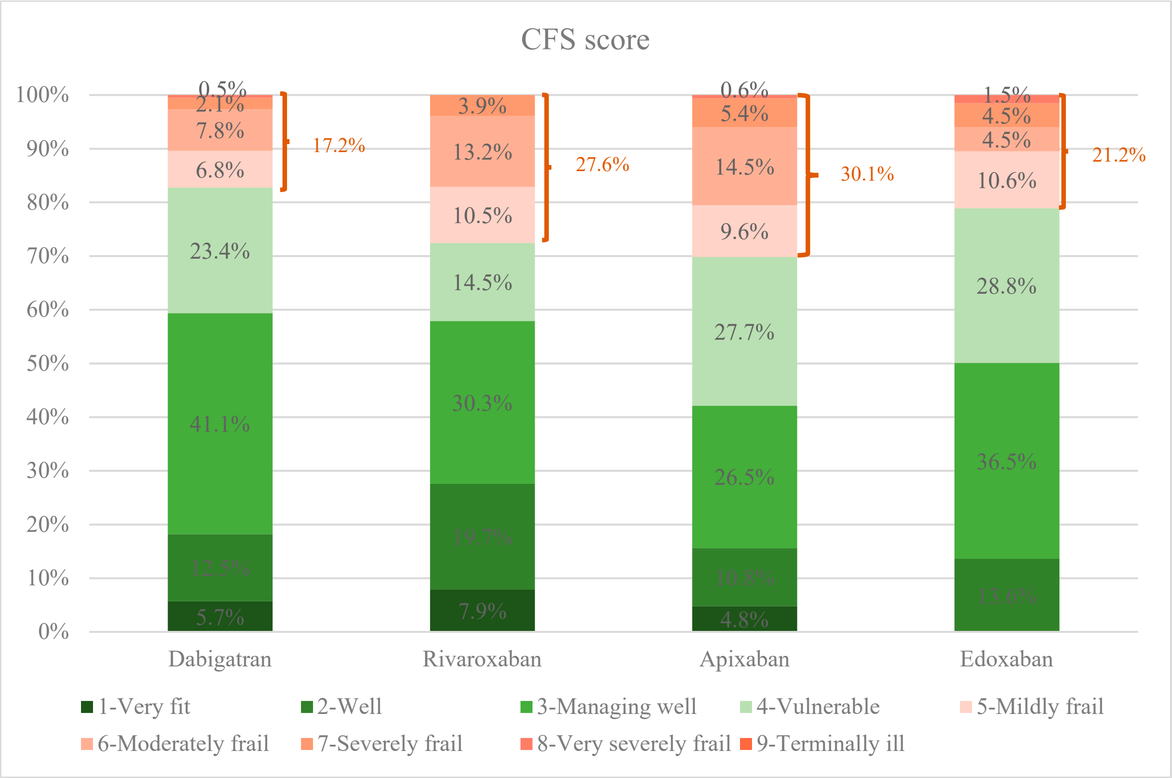

Figure S1. Clinical Frailty Scale score at the time of the study visit according to current NOAC type. CFS=Clinical frailty scale. Patients were assigned a score between 1–9 (1: very fit; 2: well; 3: managing well; 4: vulnerable; 5: mildly frail; 6: moderately frail; 7: severely frail; 8: very severely frail and 9: terminally ill) based on the evaluation of their symptoms, level of inactivity, exhaustion and basic or instrumental activities of daily living. Frailty was defined when CFS >4.

Table S2. Current NOAC treatment dose by sex

		**Male** (*n*=250)	**Female** (*n*=250)	**Total** 500
Dabigatran dose	Valid N	115	77	192
	110 mg BID	56 (48.7%)	41 (53.2%)	97 (50.5%)
	150 mg BID	59 (51.3%)	36 (46.8%)	95 (49.5%)
Rivaroxaban dose	Valid N	30	46	76
	15 mg QD	8 (26.7%)	23 (50.0%)	31 (40.8%)
	20 mg QD	22 (73.3%)	23 (50.0%)	45 (59.2%)
Apixaban dose	Valid N	72	94	166
	2.5 mg BID	31 (43.1%)	43 (45.7%)	74 (44.6%)
	5 mg BID	41 (56.9%)	51 (54.3%)	92 (55.4%)
Edoxaban dose	Valid N	33	33	66
	30 mg QD	13 (39.4%)	18 (54.5%)	31 (47.0%)
	60 mg QD	20 (60.6%)	15 (45.5%)	35 (53.0%)

BID: twice a day; QD: once a day
